# A two-stage filter for removing salt-and-pepper noise using noise detector based on characteristic difference parameter and adaptive directional mean filter

**DOI:** 10.1371/journal.pone.0205736

**Published:** 2018-10-26

**Authors:** Hongjin Ma, Yufeng Nie

**Affiliations:** School of Science, Northwestern Polytechnical University, Xi’an, 710129, China; Nanjing University of Information Science and Technology, CHINA

## Abstract

In this paper, a two-stage filter for removing salt-and-pepper noise using noise detector based on characteristic difference parameter and adaptive directional mean filter is proposed. The first stage firstly detects the noise corrupted pixels by combining characteristic difference parameter and gray level extreme, then develops an improved adaptive median filter to firstly restore them. The second stage introduces a restoration scheme to further restore the noise corrupted pixels, which firstly divides them into two types and then applies different restoration skills for the pixels based on the classification result. One type of pixels is restored by the mean filter and the other type of pixels is restored by the proposed adaptive directional mean filter. The new filter firstly adaptively selects the optimal filtering window and direction template, then replaces the gray level of noise corrupted pixel by the mean value of pixels on the optimal template. Experimental results show that the proposed filter outperforms many existing main filters in terms of noise suppression and detail preservation.

## 1 Introduction

Digital images can be corrupted by impulse noise due to bit errors in the process of image acquisition and transmission [1–3]. Salt-and-pepper noise and random-valued noise are the two common types of impulse noise [4]. The salt-and-pepper noise can corrupt an image where each corrupted pixel takes either the maximum or minimum gray level. The salt-and-pepper noise can not only significantly deteriorate the quality of an image, but also bring some difficulty to the subsequent image analysis such as image segmentation [5], edge detection [6] and classification [7, 8]. The goal of image denoising is removing noise as much as possible meanwhile preserving more edges and details. Hence, how to effectively remove the salt-and-pepper noise from corrupted images, keeps to be an important research task in image processing.

During the past several decades, many techniques have been developed to restore the corrupted image, such as the blind denoising method [9, 10] and total-variation denoising method [11, 12]. Among the traditional denoising methods, the standard median filter is one of the most popular nonlinear filters for the removal of salt-and-pepper noise in terms of its good denoising capability and computational efficiency [13]. The standard median filter uniformly replaces the gray level of each noise corrupted pixel by the median gray level of its neighborhood pixels. Hence, the standard median filter is effective in the case of low noise density. While the noise density is higher than 50%, the size of filtering window should to be enlarged to suppress serious noise which leads edges and details to be blurred. In order to improve noise suppression and detail preservation simultaneously, many modified median filters have been developed, such as the weighted median (WM) filter [14, 15], the adaptive median (AM) filter [16, 17] and the switching median (SM) filter [18–20].

The weighted median (WM) filter [14] is developed to improve the standard median filter by introducing the weighted technique. For all pixels in an image, the WM filter selects various weights for pixels at different positions in the window to restore the gray level of central pixel. Hence, the WM filter can achieve the better restoration result than the standard median filter. Different from the WM filter, the adaptive median (AM) filter [16] chooses the median gray level in an adaptive window for each pixel which overcomes the drawback of fixed window in standard median filter. Therefore, the AM filter can perform better than the standard median filter. The switching median (SM) filter [20] is a popular type of salt-and-pepper noise removing technique in recent years. The SM filter partitions the filtering process into two steps: noise detection and noise restoration. In the first step, the noise corrupted pixels are distinguished from the noise-free ones based on the noise detector. After noise detection, the gray level of each noise corrupted pixel will be replaced by the median gray level of its neighborhood pixels. The SM filter has a major drawback that the size of filtering window is fixed in the noise detection stage, which cannot effectively detect all noise corrupted pixels. In order to improve the image restoration result, many modified filters have been developed [21–23].

The modified decision based unsymmetric trimmed median (MDBUTM) [24] filter is proposed for removing salt-and-pepper noise which detects the noise corrupted pixels based on the gray level extreme and adaptively replaces the gray level of noise corrupted pixel by the mean or median variant according to the number of gray level extreme in the filtering window. The switching median filter with boundary discriminative noise detection (BDND) [25] is proposed to identify all noise corrupted pixels by adaptively selecting the filtering window of suitable size. The directional weighted median (DWM) filter [26] firstly takes into account the neighborhood information of each pixel along four directions, then uses the minimum sun of directional weighted gray level differences to detects the noise corrupted pixels, finally replaces the gray level of each noise corrupted pixel by the weighted median gray level of its neighboring pixels. The modified directional weighted median (MDWM) filter [27] is developed based on the DWM filter, which considers the neighborhood information of each pixel on more edge directions and selects the weighted median gray level excluding gray level extremes on the optimal direction as the restored gray level of the noise corrupted pixel. After deeply analyzing the DWM filter and MDWM filter, the modified directional weighted (MDW) filter [28] is proposed which firstly detects the noise corrupted pixels based on the directional gray level difference and gray level extreme, then restores each noise corrupted pixel by the weighted mean gray level of the neighborhood pixels. Besides, many other good filters are proposed in recent years. The adaptive iterative fuzzy (AIF) filter [29] is developed to restore the high density noise corrupted image which firstly detects the noise corrupted pixels with an adaptive fuzzy detector and then restores the noise corrupted pixels by a weighted mean filter. The three-values-weighted (TVW) approach [30] is proposed to restore the corrupted image, which firstly employs a variable-size local window to analyze each pixel with extreme values and classifies the non-extreme pixel as the maximum, the middle, or the minimum groups in the local window, then employs the ratios of these three groups to weight the non-extreme pixels, finally replaces the gray level of central pixel by the weighted gray level. The adaptive Type-2 fuzzy (ATF) approach [31] is proposed to remove salt-and-pepper noise which identifies the pixels based on their primary membership function values and then restores the noise corrupted pixels based on the detection results.

After deeply analyzing the performance of the existing main filters appeared in the literatures, we can find two disadvantages of their denoising schemes. One disadvantage is that most of them fail to perform well in the case of various noise densities. The other one is that although they can remove the salt-and-pepper noise from the image corrupted by high noise density, they still lead some important edges and details to be blurred. For more details, you can check the corresponding experimental results in section 3. In order to overcome the drawbacks, we propose a new two-stage filter for removing salt-and-pepper noise using noise detector based on characteristic difference parameter and adaptive directional mean filter in this paper. The proposed filter can effectively and accurately identify the noise corrupted pixels by combining the characteristic difference parameter and gray level extreme. After noise detection, the noise corrupted pixels are firstly restored by the improved adaptive median filter while the noise-free pixels are left unchanged to maintain image features. The second restoration scheme can achieve the better restoration result by proposing the adaptive directional mean filter. Compared with the above introduced filters, the proposed filter can not only effectively and accurately detect the salt-and-pepper noise, but also provide better image restoration performance in the case of various noise densities.

The other parts of this paper are organized as follows. In Section 2, we introduce the proposed method framework. Section 3 provides a number of experimental results to demonstrate the performance of the new method. Conclusions are finally drawn in Section 4.

## 2 The proposed filter

**Fig 1:**
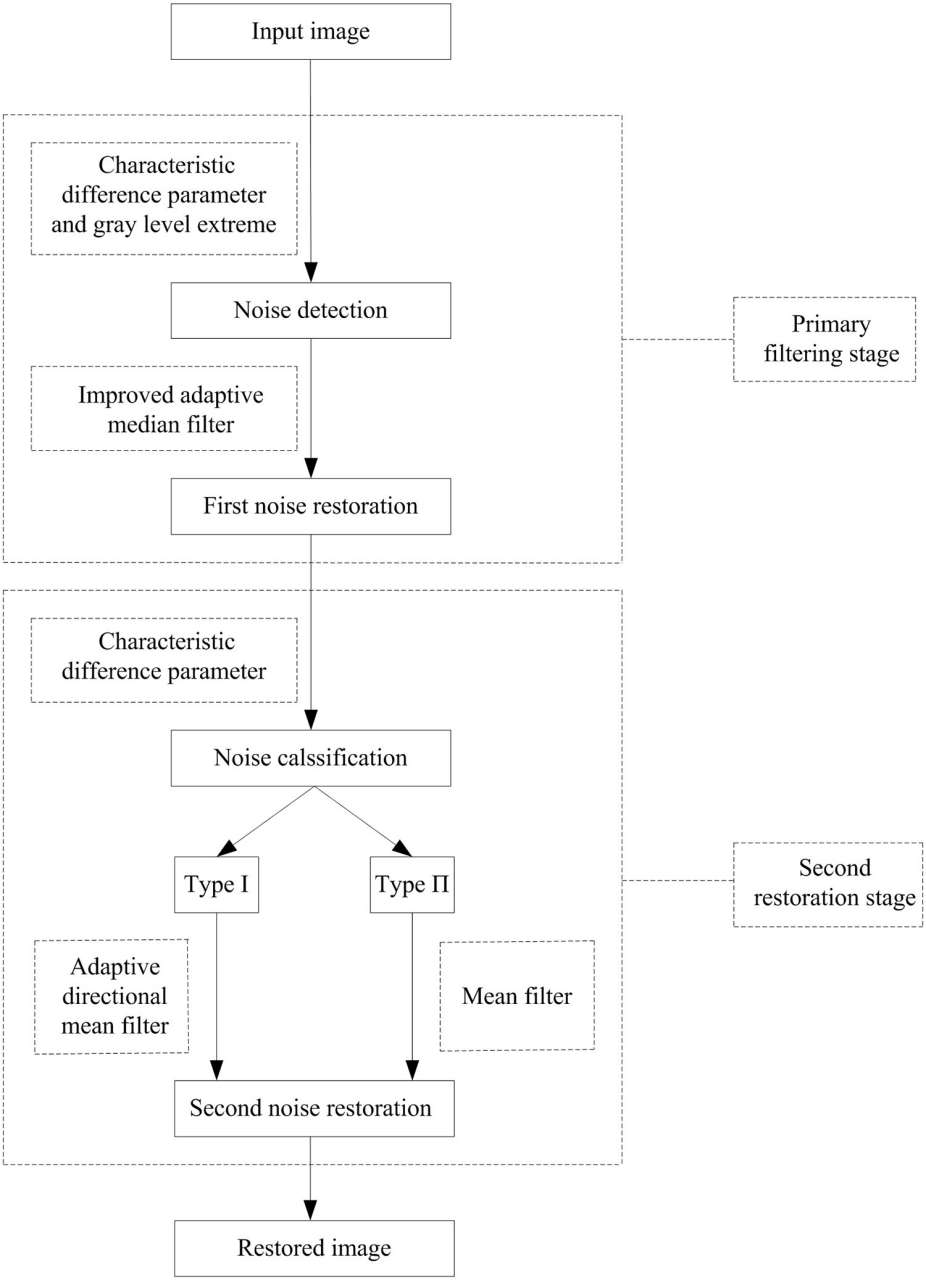
Flow chart of the proposed filter.

The proposed filter is composed of two stages: primary filtering stage and second restoration stage. The primary filtering stage firstly detects the noise corrupted pixels by combining the characteristic difference parameter and gray level extreme and then restores them by the improved adaptive median filter. In order to achieve the better restoration result, the second stage introduces a restoration scheme to secondly restore the gray levels of noise corrupted pixels. The restoration scheme firstly divides all the noise corrupted pixels into two types based on the value of characteristic difference parameter and then applies two different restoration skills for the pixels based on the classification result. And one type of pixels is secondly restored by the mean filter while the other type of pixels is secondly restored by the proposed adaptive directional mean filter. Fig 1 is the flow chart of the proposed filter.

### 2.1 Primary filtering stage

#### 2.1.1 Noise detection

In an image corrupted by salt-and-pepper noise, the gray level of noise corrupted pixel takes either the maximum or minimum gray level. Hence, the traditional switching median filter identifies the pixel with the maximum or minimum gray level as a noise corrupted pixel. However, the pixels with gray level extreme are not all the noise corrupted pixels. Therefore, the noise detector based on the judgement of gray level extreme cannot accurately detect the noise corrupted pixels. In order to achieve the better detection result, the proposed filter designs an improved noise detector by combining the characteristic difference parameter and gray level extreme.

Before introducing the characteristic difference parameter, the gray level difference is firstly introduced to compare the gray levels of two pixels. For any one pixel *P*_*i*,*j*_ in a corrupted image, the gray level difference *G*_*k*_ between the pixel *P*_*i*,*j*_ and its neighborhood pixel $P_{s_{k},t_{k}}$ is calculated as follows:

$$\begin{array}{c} G_{k}=\frac{\left|g_{i,j}-g_{s_{k},t_{k}}\right|}{g_{s_{k},t_{k}}} \end{array}$$ (1)

where (*s*_*k*_, *t*_*k*_) ∈ Ω, Ω denotes a detection window whose radius is *w*. *g*_*i*,*j*_ and $g_{s_{k},t_{k}}$ denote the gray level of pixel *P*_*i*,*j*_ and $P_{s_{k},t_{k}}$, respectively.

After that, a parameter *T* is selected as the threshold to judge the value of gray level difference. Considering that the parameter has a close relation with the corrupted image, the value of the parameter is varying based on various images. And in the proposed filter, the parameter is selected based on the statistical result for the gray levels of all pixels in the corrupted image. Besides, the size of the detection window is a key factor of affection on noise detection performance. The size of the detection window should be small in the case of low noise density but be large in the case of high noise density. Hence, the detection windows with different sizes are used for the corrupted image. After a number of experiments, it can be seen that the sizes of 3 × 3 and 5 × 5 are suitable while the noise density is lower than 50%; while the noise density locates between 60% and 80%, size of 7 × 7 is usually the best choice, and the size of 9 × 9 is suitable for the 90% noise density. Then the gray level difference between central pixel *P*_*i*,*j*_ and its neighborhood pixel $P_{s_{k},t_{k}}$ can be judged by comparing the values of *G*_*k*_ and *T* as follows:

1. If *G*_*k*_ > *T*, it means that the gray level difference between pixel *P*_*i*,*j*_ and $P_{s_{k},t_{k}}$ is large.
2. If *G*_*k*_ ≤ *T*, it means that the gray level difference between pixel *P*_*i*,*j*_ and $P_{s_{k},t_{k}}$ is small.

Based on the above analysis, it can be judged that whether the gray level difference between central pixel and each neighborhood pixel in the detection window is large. However, the comprehensive gray level difference between central pixel and all neighborhood pixels in the detection window cannot be obtained. Hence, a characteristic difference parameter is introduced to solve the problem. For any one pixel *P*_*i*,*j*_ in the corrupted image, its characteristic difference parameter *C*_*i*,*j*_ is calculated as follows:

1. According to formula (1), calculate the gray level difference *G*_*k*_ between central pixel *P*_*i*,*j*_ and its neighborhood pixel $P_{s_{k},t_{k}}$ in the detection window one by one.
2. Compare the values of gray level difference *G*_*k*_ and threshold *T*, if *G*_*k*_ > *T*, the characteristic difference parameter
  $$\begin{array}{c} C_{i,j}=C_{i,j}+1 \end{array}$$ (2)

The characteristic difference parameter can reflect the comprehensive gray level difference between the central pixel and its neighborhood pixels in the detection window, which can be used to judge that whether the central pixel is a noise corrupted pixel. The detailed analysis is given as follows:

1. If pixel *P*_*i*,*j*_ is an edge pixel, the gray level of pixel *P*_*i*,*j*_ is close to the gray levels of its neighborhood pixels on the edge direction but different from the gray levels of other neighborhood pixels in the detection window. Then the gray level difference between central pixel and its neighborhood pixel on the edge direction is small and less than the value of threshold *T*. But the gray level difference between central pixel and other neighborhood pixels is large and greater than the value of threshold *T*. Since the number of neighborhood pixels on the edge direction is less than the number of other neighborhood pixels, the characteristic difference parameter *C*_*i*,*j*_ is large. However, considering that the number of neighborhood pixels on the edge direction is at least 2*w*, the characteristic difference parameter *C*_*i*,*j*_ is less than or equal to 2*w*(2*w* + 1).
2. If pixel *P*_*i*,*j*_ is a noise-free pixel in a flat region, the gray levels of all pixels in the detection window are close. Then the gray level difference between central pixel and each neighborhood pixel is much small and less than the value of threshold *T*. Hence, the value of characteristic difference parameter *C*_*i*,*j*_ is small and less than 2*w*(2*w* + 1).
3. If the central pixel *P*_*i*,*j*_ is a noise corrupted pixel located on an edge, the gray level of pixel *P*_*i*,*j*_ is much different from the gray levels of its neighborhood pixels in the detection window while the gray levels of pixels on the edge direction are close. But the gray level difference between central pixel and each neighborhood pixel is large and greater than the value of threshold *T*. Hence, the characteristic difference parameter *C*_*i*,*j*_ is large and greater than 2*w*(2*w* + 1).
4. If pixel *P*_*i*,*j*_ is a noise corrupted pixel in a flat region, the gray level of pixel *P*_*i*,*j*_ is much different from the gray levels of its neighborhood pixels in the detection window while the gray levels of its neighborhood pixels are close. Then the gray level difference between central pixel and each neighborhood pixel is large and greater than the value of threshold *T*. Hence, the characteristic difference parameter *C*_*i*,*j*_ is large and greater than 2*w*(2*w* + 1).

From the above analysis, it can be learned that the characteristic difference parameter can be used to distinguish noise corrupted pixels from noise-free ones in a corrupted image. And the threshold is selected to judge the value of characteristic difference parameter. The value of the threshold to is equal to the number of all the pixels except for the pixels on the diagonal in the detection window. Then the proposed filter can effectively and accurately detect the noise corrupted pixels by combining characteristic difference parameter and gray level extreme. And for any one pixel *P*_*i*,*j*_, its characteristic can be identified in the following way:

$$\begin{array}{c} P_{i,j}\;\in \{\begin{array}{cc} N, & if\;C_{i,j}>2w\left(2w+1\right)\;and\;g_{i,j}\in \{0,255\}\\ S, & otherwise \end{array} \end{array}$$ (3)

where *N* and *S* denote the noise corrupted pixels and noise-free ones, respectively. *w* is the radius of the detection window. *g*_*i*,*j*_ is the gray level of pixel *P*_*i*,*j*_.

The pseudo codes of the process of noise detection are provided as follows:

For each pixel *P*_*i*,*j*_ in the corrupted image:

 *C*_*i*,*j*_ = 0.

 **For** $P_{s_{k},t_{k}}$ in Ω.

   $G_{k}=\frac{\left|g_{i,j}-g_{s_{k},t_{k}}\right|}{g_{s_{k},t_{k}}}$.

   if *G*_*k*_ > *T*

    *C*_*i*,*j*_ = *C*_*i*,*j*_ + 1.

 **end**

 **if** *C_i,j_* > 2*w* (2*w* + 1) *and g_i,j_* ∈ {0,255}

  *P*_*i*,*j*_ ∈ *N*.

 **else**

  *P*_*i*,*j*_ ∈ *S*.

 **end**

**end**

#### 2.1.2 First noise restoration

After noise detection, an improved adaptive median filter is introduced to firstly restore the gray levels of the noise corrupted pixels. And for each noise corrupted pixel, its gray level is replaced by the mean value of the gray levels of its neighboring pixels in the adaptive window. However, the noise-free ones are left unchanged to maintain the features of the original image. The primary filtering process is given as follows:

1. For any one pixel *p*_*i*,*j*_ in the corrupted image, select a detection window of suitable size with a small radius *w* and set the maximum radius of the detection window to be *w*_max_.
2. Compare $S_{i,j}^{\max,w}$, $S_{i,j}^{\min,w}$ and $S_{i,j}^{med,w}$, which are the maximum value, minimum value and median value among the gray levels of the pixels in the detection window, respectively.
3. If $S_{i,j}^{\min,w}<S_{i,j}^{med,w}<S_{i,j}^{\max,w}$, then go to the step 5) and calculate the characteristic difference parameter *C*_*i*,*j*_; Otherwise, *w* = *w* + 1.
4. If *w* ≤ *w*_max_, go to the step 2); Otherwise, the restoration gray level of the pixel $g_{i,j}^{1}=S_{i,j}^{med}(w_{\max})$, and stop.
5. If $\left(g_{i,j}=S_{i,j}^{\max,w}|g_{i,j}=S_{i,j}^{\min,w}\right)$ and *C*_*i*,*j*_ > 2*w*(2*w* + 1), the pixel *p*_*i*,*j*_ is corrupted, and the restoration gray level of the pixel $g_{i,j}^{1}=S_{i,j}^{med}(w)$; Otherwise, the pixel *p*_*i*,*j*_ is uncorrupted, $g_{i,j}^{1}=g_{i,j}$, and stop.

On this basis, according to the result of noise detection the primary restoration gray level $g_{i,j}^{1}$ of the pixel *p*_*i*,*j*_ is calculated as follows:

$$\begin{array}{c} g_{i,j}^{1}=\{\begin{array}{cc} g_{i,j}, & p_{i,j}\in S\\ imadpmed(g_{i,j}), & p_{i,j}\in N \end{array} \end{array}$$ (4)

where *imadpmed*() denotes the improved adaptive median filter.

### 2.2 Second restoration stage

#### 2.2.1 Noise classification

Since the improved adaptive median filter cannot restore the corrupted image well in the case of high noise density, the second stage introduces a restoration scheme to secondly restore the gray levels of noise corrupted pixels. Considering that the characteristic difference parameter can reflect the comprehensive gray level difference between each pixel and its neighborhood pixels in a local window, then before the second restoration, the restoration scheme firstly divides all the noise corrupted pixels into two types based on the value of characteristic difference parameter in a 3 × 3 window. And then according to the classification result two different restoration skills are used to secondly restore the noise corrupted pixels. Besides, the gray levels of noise-free pixels are still left unchanged to maintain image features.

Refer to the calculation process of the characteristic difference parameter in the previous section, for the noise corrupted pixel *p*_*i*,*j*_, its characteristic difference parameter $C_{i,j}^{3}$ in the 3 × 3 window Ω_3_ centered at the position (*i*, *j*) is calculated as follows:

1. According to the formula (1), calculate the gray level difference $G_{k}^{3}$ between central pixel *p*_*i*,*j*_ and its neighborhood pixel $p_{s_{k},t_{k}}$ in the window Ω_3_ one by one.
2. Compare the values of gray level difference $G_{k}^{3}$ and threshold *T*, if $G_{k}^{3}>T$, the characteristic difference parameter $C_{i,j}^{3}=C_{i,j}^{3}+1$.

Since the radius of the 3 × 3 window is 1, the threshold for characteristic difference parameter $C_{i,j}^{3}$ is selected to be 6. Then based on the value of characteristic difference parameter $C_{i,j}^{3}$, the comprehensive gray level difference between the noise corrupted pixel *p*_*i*,*j*_ and its neighborhood pixels after primary filtering is judged as follows:

1. If $C_{i,j}^{3}\leq 6$, it means that after primary filtering the gray level of noise corrupted pixel *p*_*i*,*j*_ is not much different from the gray levels of its neighborhood pixels.
2. If $C_{i,j}^{3}>6$, it means that after primary filtering the gray level of noise corrupted pixel *p*_*i*,*j*_ is much different from the gray levels of its neighborhood pixels.

According to the above judgement process, the comprehensive gray level differences between each noise corrupted pixel and all the neighborhood pixels in the 3 × 3 window can be judged. And based on the judgement result, all the noise corrupted pixels after primary filtering can be divided into two types. Among all the noise corrupted pixels, the noise corrupted pixels whose characteristic difference parameters in the 3 × 3 window are greater than 6 belong to one type and the rest of the noise corrupted pixels belong to the other type.

#### 2.2.2 Second noise restoration

Based on the above noise classification result, two different restoration skills will be applied for the noise corrupted pixels. The noise corrupted pixels, whose characteristic difference parameters in the the 3 × 3 window are greater than 6, are secondly restored by the proposed adaptive directional mean filter. And the rest of the noise corrupted pixels are secondly restored by the mean filter in the 3 × 3 window. Then for any one pixel *p*_*i*,*j*_, its second restoration gray level $g_{i,j}^{2}$ is calculated according to the following different situations:

$$\begin{array}{c} g_{i,j}^{2}=\{\begin{array}{cc} g_{i,j}^{1}, & p_{i,j}\in S\\ mean_{3}(g_{i,j}^{1}), & p_{i,j}\in N\;\;and\;\;C_{i,j}^{3}\leq 6\\ adpdir(g_{i,j}^{1}), & p_{i,j}\in N\;\;and\;\;C_{i,j}^{3}>6 \end{array} \end{array}$$ (5)

where m*ean*_3_() denotes the mean filter with the filtering window of size 3 × 3. *adpdir*() is the proposed adaptive directional mean filter.

**Fig 2:**
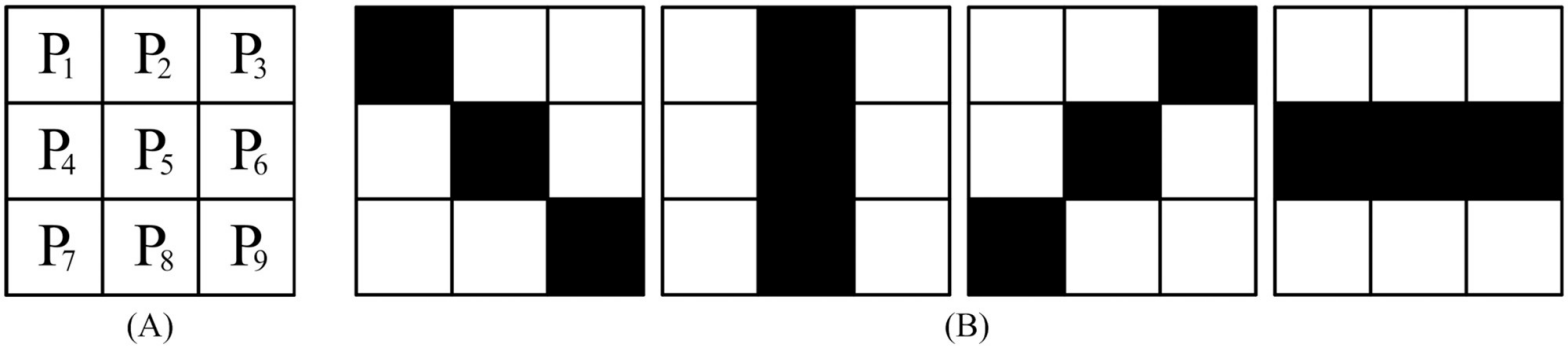
3 × 3 filtering window and four direction templates. (A) 3 × 3 filtering window. (B) Four direction templates.

The adaptive directional mean filter is developed to restore the gray levels of noise corrupted pixels based on the multi-directional image information. Firstly, the proposed filter designs four direction filtering templates in the filtering windows with different size, then adaptively selects the optimal filtering window and direction template according to the characteristics of pixels in the neighborhood, finally replaces the gray level of each noise corrupted pixel by the mean value of the gray levels of pixels on the optimal direction template. The detailed process of the adaptive directional mean filter is given as follows:

**Step 1**: Establish a filtering window of size 3 × 3 as shown in Fig 2(A) and design four direction templates as shown in Fig 2(B).

At first, it should be judged that whether the optimal direction template can be selected from the four direction templates in the 3 × 3 filtering window. And the judgement condition is that all the pixels except for the central pixel on each direction template are noise-free pixels or second restored pixels. Considering that the filtering order of the denoising method are from left to right and from top to bottom, pixels *P*_1_, *P*_2_, *P*_3_, *P*_4_ are noise-free pixels or second restored pixels. Then, only if all the pixels *P*_6_, *P*_7_, *P*_8_, *P*_9_ are noise-free pixels, the optimal direction template can be selected from the four templates in the 3 × 3 filtering window. Otherwise, the size of the filtering window need to be further enlarged as shown in the next step. The selection process of the optimal direction template is as follows:

1. Calculate the absolute gray level difference *a*^*l*^ of the pixels on every direction template *E*^*l*^ except for the central pixel as follows:
  $$\begin{array}{c} a^{l}=|g_{s_{1},t_{1}}^{l}-g_{s_{2},t_{2}}^{l}| \end{array}$$ (6)
  where *l*(1 ≤ *l* ≤ 4) is the direction index, $g_{s_{1},t_{1}}^{l}$ and $g_{s_{2},t_{2}}^{l}$ denote the gray levels of the pixels at the position (*s*_1_, *t*_1_) and (*s*_2_, *t*_2_) of the filtering template *E*^*l*^, respectively.
2. Identify the minimum gray level difference $a^{l^{*}}$ among the four absolute gray level differences as follows:
  $$\begin{array}{c} a^{l^{*}}=\min\{a^{l},1\leq l\leq 4\} \end{array}$$ (7)
3. Select the direction template with the minimum gray level difference as the optimal one, and denote it as $E^{l^{*}}$.

After selecting the optimal direction template, the noise-free pixels and the second restored pixels on the optimal direction template are selected to restore the gray level of the central pixel. And in this step, two pixels on the optimal direction template are selected to restore the gray level of the central pixel. Then the second restoration gray level $g_{i,j}^{2}$ is the mean gray level of the two pixels on the optimal direction template and it is calculated as follows:

$$\begin{array}{c} g_{i,j}^{2}=\frac{g_{s_{1},t_{1}}^{l^{*}}+g_{s_{2},t_{2}}^{l^{*}}}{2} \end{array}$$ (8)

where $g_{s_{1},t_{1}}^{l^{*}}$ and $g_{s_{2},t_{2}}^{l^{*}}$ denote the gray levels of the pixels at the position (*s*_1_, *t*_1_) and (*s*_2_, *t*_2_) of the optimal direction template $E^{l^{*}}$, respectively.

Besides, if the pixels *P*_6_, *P*_7_, *P*_8_, *P*_9_ are not all noise-free pixels, the optimal direction template cannot be selected from the four templates in the 3 × 3 window and the next step will be performed.

**Fig 3:**
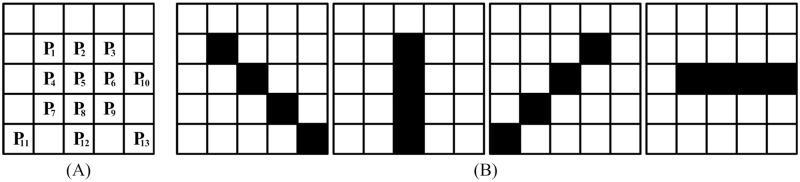
5 × 5 filtering window and four direction templates. (A) 5 × 5 filtering window. (B) Four direction templates.

**Step 2**: Enlarge the size of filtering window to be 5 × 5 as shown in Fig 3(A) and design four direction templates as shown in Fig 3(B).

Similar to the previous step, it should be judged that whether the optimal direction template can be selected from the four templates in the 5 × 5 filtering window. And the judgement condition is that except for the central pixel, there are at least two noise-free pixels or second restored pixels on each direction template. In other words, only if all the direction filtering template meet the condition, the optimal direction template can be selected from the four templates in the 5 × 5 filtering window. Otherwise, the size of filtering window need to be further enlarged as shown in the final step. From the previous step, it is known that the pixels *P*_1_, *P*_2_, *P*_3_, *P*_4_ are noise-free pixels or second restored pixels. Therefore, the characteristics of other pixels *P*_6_, *P*_7_, *P*_8_, *P*_9_, *P*_10_, *P*_11_, *P*_12_, *P*_13_ will be identified. And the first direction template as shown in Fig 3(B) is taken as an example to introduce the judgement process of pixels on the direction template as follows:

1. The pixel *P*_9_ is firstly identified whether it is a noise-free pixel. If it is a noise-free pixel, pixel *P*_1_ and pixel *P*_9_ will be selected as the pixels on the direction template. Instead, if it is a noise corrupted pixel, the characteristic of pixel *P*_13_ will be judged.
2. If the pixel *P*_13_ is a noise-free pixel, pixel *P*_1_ and pixel *P*_13_ will be selected as the pixels on the direction template. Otherwise, if it is a noise corrupted pixel, the size of filtering window need to be further enlarged to be 7 × 7.

After judging the first direction template, the other three direction templates shown in Fig 3(B) will be judged that whether they can meet the condition. For all the four direction templates, if no need to enlarge the size of filtering window appears, the filtering window of size 5 × 5 can be used to restore the gray level of the central noise corrupted pixel. And similar to the step 1, the same selection process for the optimal direction template will be performed. Similarly, in this step two pixels are selected to restore the gray level of the central pixel. And according to the same calculated process as the step 1, the gray level of the central pixel will be replaced by the mean gray level of the selected pixels on the optimal direction template. Instead, if the optimal direction template cannot be selected from the four direction templates in the 5 × 5 filtering window, the final step will be performed.

**Fig 4:**
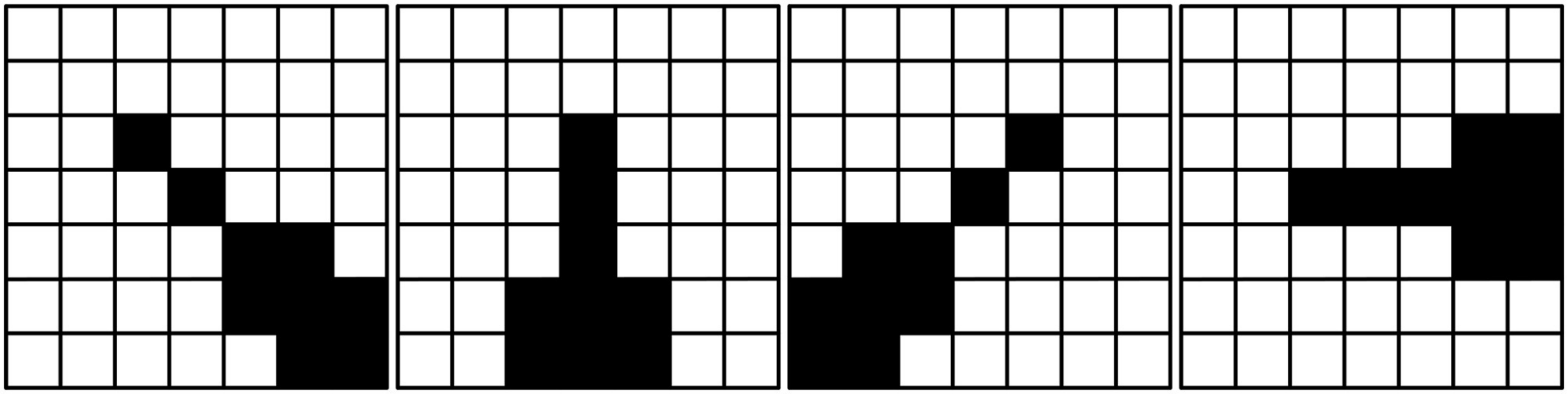
Four direction templates in 7 × 7 filtering window.

**Step 3**: Further enlarge the size of filtering window to be 7 × 7 and design four direction templates as shown in Fig 4.

From Fig 4, it can be seen that there are more pixels in every direction template in the 7 × 7 filtering window than the above two filtering windows. Hence, the filtering window will not be further enlarged up to now and the optimal direction template will be selected from the four direction templates as follows:

1. Identify all the noise-free and second corrected pixels on every direction template *E*^*l*^.
2. For each direction template *E*^*l*^, identify all the noise-free pixels and second restored pixels and then select the pixel with the maximum gray level $g_{\max}^{l}$ and the pixel with the minimum gray level $g_{\min}^{l}$ among all the noise-free pixels and second restored pixels.
3. Calculate the absolute gray level difference *a*^*l*^ of $g_{\max}^{l}$ and $g_{\min}^{l}$ on every direction template as follows:
  $$\begin{array}{c} a^{l}=|g_{\max}^{l}-g_{\min}^{l}|\;\; \end{array}$$ (9)
  where *l*(1 ≤ *l* ≤ 4) is the direction index.
4. Identify the minimum gray level difference $a^{l^{*}}$ among the four absolute gray level differences by the following formula:
  $$\begin{array}{c} a^{l^{*}}=\min\{a^{l},1\leq l\leq 4\} \end{array}$$ (10)
5. Select the direction template with the minimum gray level difference as the optimal one, and denote it as $E^{l^{*}}$.

Similar to the above steps, after selecting the optimal direction template the noise-free pixels and the second restored pixels on the optimal direction template are selected to restore the gray level of the central pixel. And the gray level of the central pixel will be replaced by the mean gray level of the selected pixels on the optimal direction template. Then the second restoration gray level $g_{i,j}^{2}$ of the central pixel is calculated according to the following formula:

gi,j2=∑k=1ngsk,tkl*/∑k=1ngsk,tkl*nn (11)

where $g_{s_{k},t_{k}}^{l^{*}}$ denotes the selected pixel on the optimal direction template. The value of *n* is equal to the number of the selected pixels.

## 3 Experimental results

In order to demonstrate the effectiveness of the proposed filter, the proposed filter is firstly compared with five representative denoising methods include the switching median (SM) filter [20], the directional weighted median (DWM) filter [26], the modified directional weighted median (MDWM) filter [27], the modified directional weighted (MDW) filter [28] and the three-values-weighted (TVW) filter [30]. Three typical gray level images with different image features include Lena, Boat and Zelda are selected as the test images. The performance of image restoration results is quantitatively evaluated by two measures, namely, PSNR (peak signal-to-noise ratio) [32] and MSSIM (mean structural similarity) [33]. The higher the value of PSNR is, the better the quality of the restored image is. PSNR can be expressed as

$$\begin{array}{c} PSNR=10\log_{10}(\frac{MAX}{MSE}) \end{array}$$ (12)

where *MAX* is the maximum gray level of an image and its value is 255 for a gray level image. The *MSE* denotes the mean square error between the original noise-free image and the restored image, which can be calculated by

$$\begin{array}{c} MSE=\frac{1}{MN}\sum_{i=0}^{M-1}\sum_{j=0}^{N-1}{\left(s_{i,j}-{\hat{s}}_{i,j}\right)}^{2} \end{array}$$ (13)

where *s*_*i*,*j*_ and ${\hat{s}}_{i,j}$ denote the original pixel and the restored pixel, respectively. *M* and *N* are the sizes of an image for the width and the height.

MSSIM reflects the structural similarity between the restored image and the original noise-free image. The higher value of MSSIM means that the structures in the restored image can be preserved more completely. MSSIM is defined as

$$\begin{array}{c} MSSIM\left(x,y\right)=\frac{1}{W}\sum_{m=1}^{W}SSIM(x_{m},y_{m}) \end{array}$$ (14)

where *x* and *y* denote the original noise-free image and the restored image, respectively. *W* is the number of local windows in the image. The *SSIM* denote the structural similarity between the original noise-free image and the restored image which is given by

$$\begin{array}{c} SSIM\left(x,y\right)=\frac{\left(2\mu_{x}\mu_{y}+C_{1})(2\sigma_{xy}+C_{2}\right)}{\left(\mu_{x}^{2}+\mu_{y}^{2}+C_{1})(\sigma_{x}^{2}+\sigma_{y}^{2}+C_{2}\right)} \end{array}$$ (15)

where *μ*_*x*_ and *μ*_*y*_ are the means of the input image and the restored image, respectively. *σ*_*xy*_ is the covariance of the input image and the restored image. $\sigma_{x}^{2}$ and $\sigma_{y}^{2}$ are the variances of the input image and the restored image, respectively. *C*_1_ and *C*_2_ are the constants which can be calculated according to the paper [24].

Table 1 presents the performance comparisons for the different filters in terms of PSNR value for the test images. It can be seen that in the case of low noise corruption (noise density less than 30%), all the filters can perform well. In the case of medium noise corruption, such as 40%-70% noise density, the MDWM, MDW, TVW filters and the proposed filter reveal the superior denoising performance then the SM and DWM filters. As to the cases of heavy noise corruptions (noise density higher than 80%), the TVW filter and the proposed filter achieve the better results than the SM, DWM, MDWM and MDW filters. And compared to other filters, the proposed filter can obtain the higher value of PSNR in the cases of various noise densities. Hence, Table 1 indicates that the proposed filter is able to more effectively remove salt-and-pepper noise than other compared filters.

**Table 1:** Comparisons of restoration results in PSNR value for the test images.

Image	Noise density(%)	Denoising algorithms					
		SM	DWM	MDWM	MDW	TVW	Proposed
Lena	10	36.12	40.78	41.50	42.71	42.53	43.08
	20	33.42	37.02	38.13	39.49	39.12	39.87
	30	31.36	34.63	36.10	37.28	36.92	37.63
	40	29.88	32.51	34.16	35.41	35.16	35.79
	50	28.54	30.23	32.62	33.44	33.87	34.02
	60	26.76	27.69	31.22	31.34	32.29	32.13
	70	24.47	25.23	29.77	30.35	30.95	31.10
	80	19.52	21.00	27.94	28.81	29.12	29.45
	90	8.80	15.45	25.34	26.57	26.84	27.16
Boat	10	32.84	36.19	38.60	39.78	39.48	39.98
	20	30.05	32.81	35.41	36.53	36.31	36.85
	30	28.15	30.44	33.15	34.21	34.02	34.42
	40	26.76	28.61	31.31	32.60	32.54	32.91
	50	25.08	26.70	29.82	30.85	31.14	31.30
	60	23.16	24.25	28.32	28.15	29.55	28.99
	70	20.54	22.10	26.85	27.20	28.15	28.16
	80	13.25	18.36	25.09	25.94	26.40	26.67
	90	7.83	14.42	22.73	23.80	23.98	24.38
Zelda	10	39.72	43.39	45.49	47.26	46.73	47.61
	20	36.90	39.85	42.04	43.84	43.47	44.15
	30	35.26	37.76	40.00	41.56	41.22	41.97
	40	33.48	35.27	37.93	39.53	39.37	40.18
	50	31.98	33.32	36.39	36.24	37.85	37.42
	60	30.05	30.92	34.79	34.96	36.25	36.28
	70	25.79	27.26	33.05	33.81	34.57	34.74
	80	14.55	22.85	31.12	31.87	32.83	32.99
	90	8.42	16.33	28.35	29.41	30.27	30.52

The performance comparisons for the different filters in terms of MSSIM value for the test images are shown in Table 2. One can observe that the MSSIM values of the restoration results of the SM and DWM filters are significantly less than the MDWM, MDW, TVW filters and the proposed filter in the case of various noise densities. It means that the MDWM, MDW, TVW filters and the proposed filter have stronger capability than the SM and DWM filters for preserving image structures. Besides, in the case of higher noise density, the TVW filter and the proposed filter can achieve the larger values of MSSIM than the MDWM and MDW filters. This indicates that the TVW filter and the proposed filter can preserve image structures better than the MDWM and MDW filters at heavy noise density. In addition, the proposed filter achieves the largest value of MSSIM among the different filters in the case of various noise densities. In other words, the structures in the restoration results of the proposed filter can be preserved more completely than other compared filters.

**Table 2:** Comparisons of restoration results in MSSIM value for the test images.

Image	Noise density(%)	Denoising algorithms					
		SM	DWM	MDWM	MDW	TVW	Proposed
Lena	10	0.9934	0.9908	0.9938	0.9975	0.9975	0.9976
	20	0.9972	0.9739	0.9868	0.9943	0.9943	0.9955
	30	0.9634	0.9511	0.9778	0.9902	0.9904	0.9921
	40	0.9431	0.9203	0.9643	0.9836	0.9848	0.9877
	50	0.9214	0.8772	0.9481	0.9678	0.9781	0.9801
	60	0.8890	0.8178	0.9263	0.9560	0.9682	0.9700
	70	0.8094	0.7328	0.8951	0.9407	0.9544	0.9616
	80	0.6112	0.5865	0.8462	0.9065	0.9268	0.9397
	90	0.2402	0.3816	0.7568	0.4223	0.8702	0.9007
Boat	10	0.9727	0.9868	0.9932	0.9976	0.9975	0.9976
	20	0.9501	0.9625	0.9851	0.9941	0.9941	0.9945
	30	0.9161	0.9307	0.9736	0.9887	0.9891	0.9894
	40	0.8713	0.8882	0.9590	0.9818	0.9835	0.9839
	50	0.8189	0.8306	0.9385	0.9646	0.9753	0.9754
	60	0.7506	0.7515	0.9112	0.9390	0.9639	0.9507
	70	0.6519	0.6427	0.8695	0.9151	0.9462	0.9461
	80	0.4703	0.5007	0.8078	0.8734	0.9112	0.9116
	90	0.2376	0.3184	0.7012	0.3622	0.8341	0.8352
Zelda	10	0.9916	0.9954	0.9965	0.9991	0.9991	0.9992
	20	0.9824	0.9874	0.9923	0.9978	0.9978	0.9980
	30	0.9700	0.9745	0.9867	0.9960	0.9962	0.9964
	40	0.9563	0.9558	0.9783	0.9924	0.9937	0.9940
	50	0.9348	0.9266	0.9673	0.9794	0.9905	0.9907
	60	0.9062	0.8805	0.9515	0.9749	0.9857	0.9858
	70	0.8338	0.7882	0.9245	0.9632	0.9772	0.9773
	80	0.5934	0.6275	0.8895	0.9390	0.9613	0.9617
	90	0.1165	0.3673	0.8103	0.8891	0.9198	0.9206

Tables 3 and 4 present the quantitative comparisons for the various filters in terms of the average PSNR value and the average MSSIM value for the three test images, respectively. According to Table 3, one can observe that the MDWM, MDW, TVW filters and the proposed filter achieve the larger average PSNR value than the SM and DWM filters in the cases of various noise densities. In the heavy noise corruption, the TVW filter and the proposed filter can perform better than the MDWM and MDW filters. And the proposed filter usually obtains the largest average PSNR value. From the quantitative comparisons as shown in Table 4, we can obtain the similar conclusions. Hence, the proposed filter can achieves the larger average values of PSNR and MSSIM than other filters which can indicate that the proposed filter outperforms than the other filters in image denoising and detail preservation.

**Table 3:** Quantitative comparisons of restoration results in average PSNR value for the test images.

Noise density(%)	Denoising algorithms					
	SM	DWM	MDWM	MDW	TVW	Proposed
10	36.23	40.12	41.86	43.25	42.91	43.56
20	33.46	36.56	38.53	39.95	39.63	40.29
30	31.59	34.28	36.42	37.68	37.39	38.01
40	30.04	32.13	34.47	35.85	35.69	36.29
50	28.53	30.08	32.94	33.51	34.29	34.30
60	26.66	27.62	31.44	31.48	32.70	32.47
70	23.26	24.86	29.89	30.45	31.22	31.33
80	15.77	20.74	28.05	28.87	29.45	29.70
90	8.35	15.40	25.47	26.59	27.03	27.35

**Table 4:** Quantitative comparisons of restoration results in average MSSIM value for the test images.

Noise density(%)	Denoising algorithms					
	SM	DWM	MDWM	MDW	TVW	Proposed
10	0.9859	0.9910	0.9945	0.9981	0.9980	0.9981
20	0.9766	0.9746	0.9881	0.9954	0.9954	0.9960
30	0.9498	0.9521	0.9794	0.9916	0.9919	0.9926
40	0.9236	0.9214	0.9672	0.9859	0.9873	0.9885
50	0.8917	0.8781	0.9513	0.9706	0.9813	0.9820
60	0.8486	0.8166	0.9297	0.9566	0.9726	0.9688
70	0.7650	0.7212	0.8964	0.9397	0.9593	0.9617
80	0.5583	0.5716	0.8478	0.9063	0.9331	0.9377
90	0.1981	0.3558	0.7561	0.5579	0.8747	0.8855

**Fig 5:**
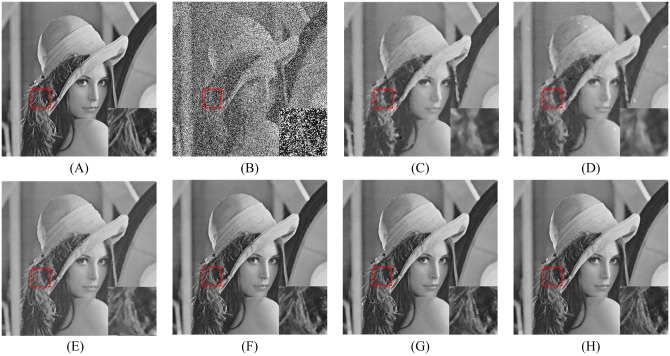
Restoration results of various filters for the Lena image with 50% noise density. (A) Noise-free image. (B) Noise corrupted image. (C) Restoration result of SM filter. (D) Restoration result of DWM filter. (E) Restoration result of MDWM filter. (F) Restoration result of MDW filter. (G) Restoration result of TVW filter. (H) Restoration result of proposed filter.

**Fig 6:**
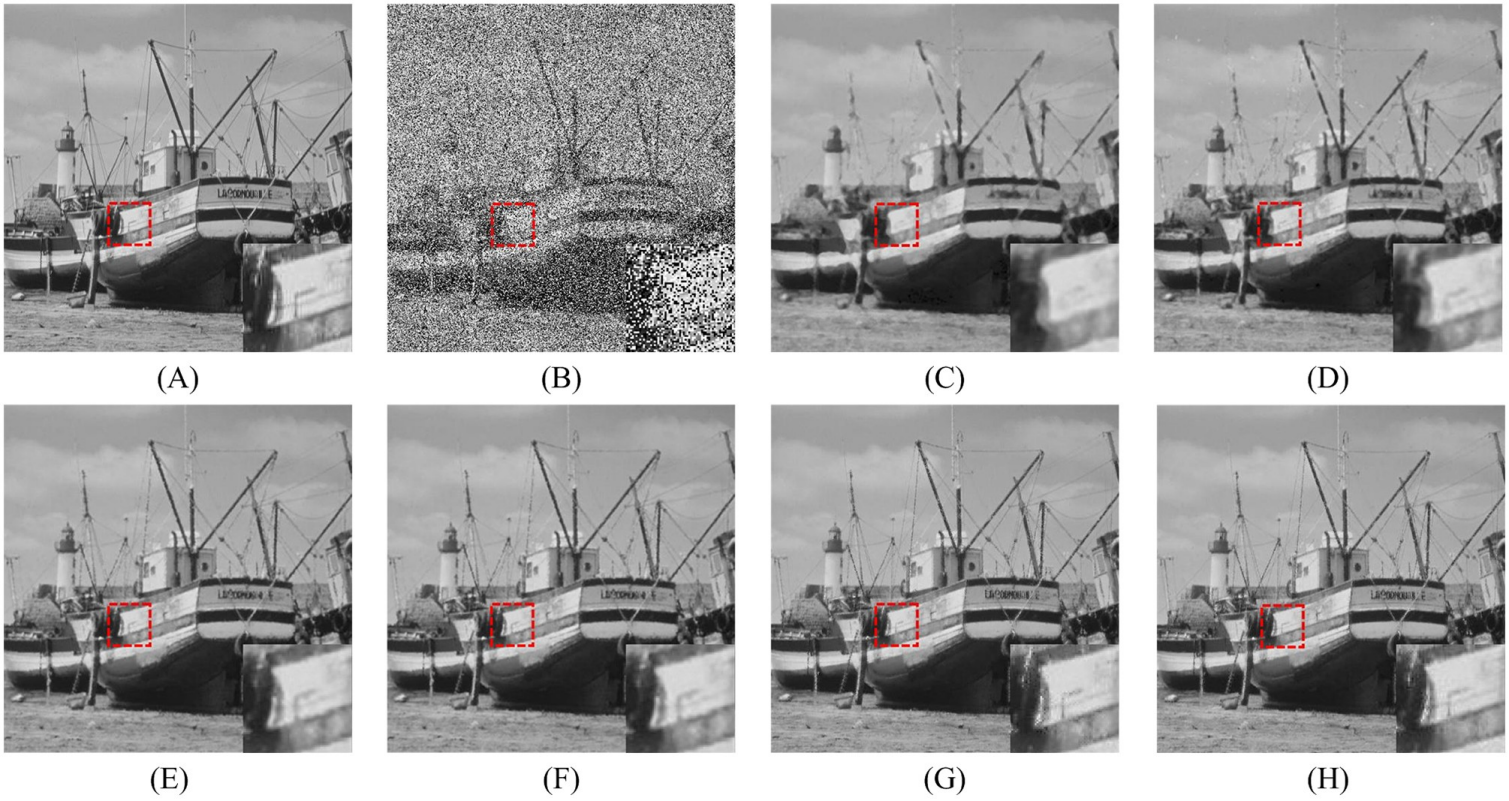
Restoration results of various filters for the Boat image with 50% noise density. (A) Noise-free image. (B) Noise corrupted image. (C) Restoration result of SM filter. (D) Restoration result of DWM filter. (E) Restoration result of MDWM filter. (F) Restoration result of MDW filter. (G) Restoration result of TVW filter. (H) Restoration result of proposed filter.

**Fig 7:**
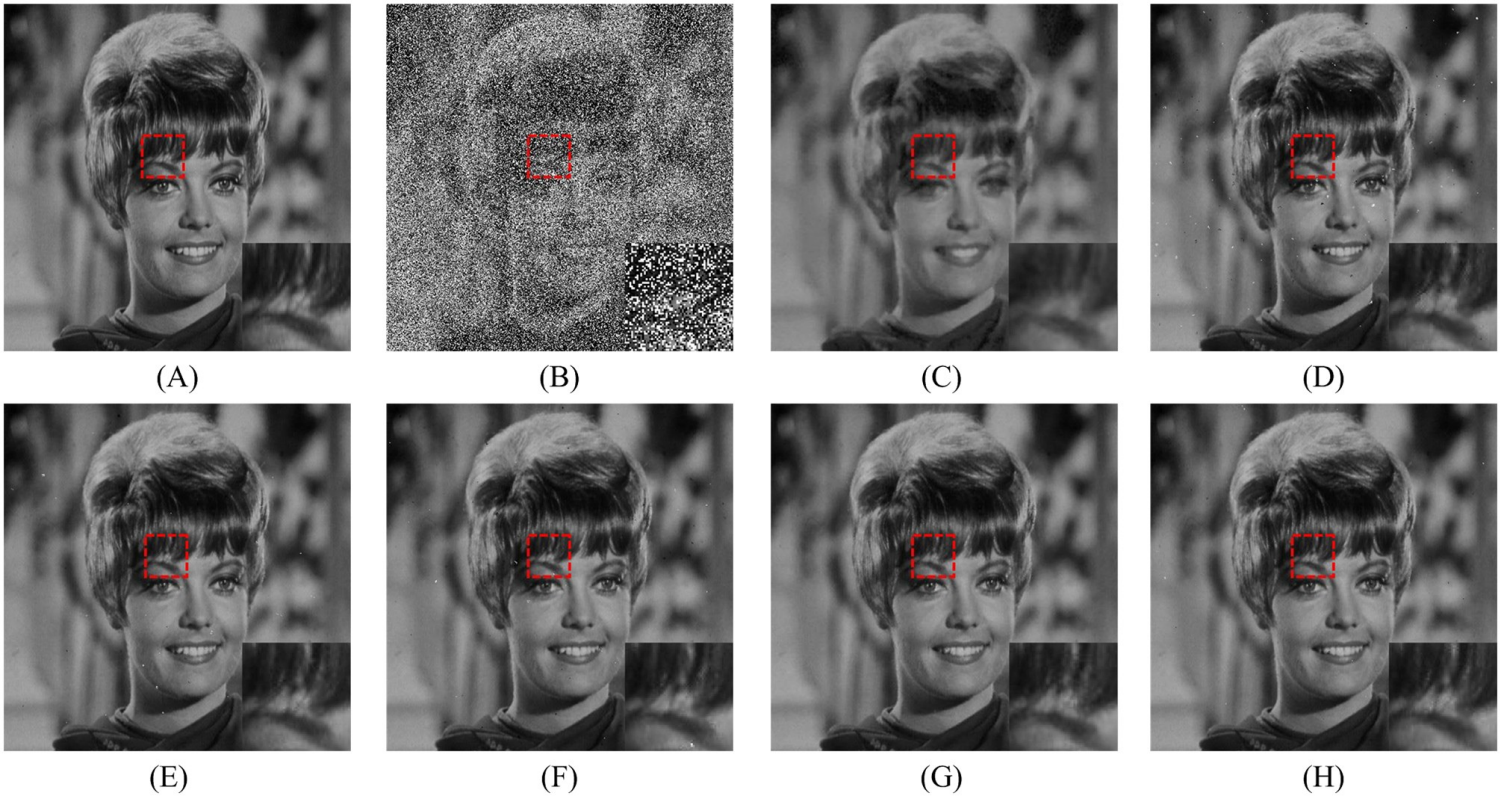
Restoration results of various filters for the Zelda image with 50% noise density. (A) Noise-free image. (B) Noise corrupted image. (C) Restoration result of SM filter. (D) Restoration result of DWM filter. (E) Restoration result of MDWM filter. (F) Restoration result of MDW filter. (G) Restoration result of TVW filter. (H) Restoration result of proposed filter.

In order to explore the visual quality of the various filters, we show the restored images in the case of different noise density. Figs 5, 6 and 7 show the restoration results of various filters for the Lena, Boat and Zelda images, respectively. And the test images are corrupted by salt-and-pepper noise with 50% noise density. One can observe that the AM and DWM filters obtain the worst image restoration effect. Although they can remove the salt-and-pepper noise from the corrupted images, they still suffer from the blurred effect for the edges and details in the restored images. The MDWM, MDW, TVW filters and the proposed filter can efficiently remove the noise and be free from the blurred effect in the restored images. Besides, the TVW filter and the proposed filter achieve the better visual effect than other filters, because they can preserve more edges and contain less noise in the restoration images.

**Fig 8:**
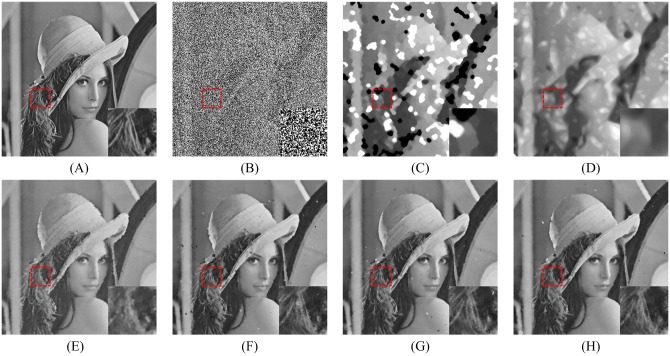
Restoration results of various filters for the Lena image with 80% noise density. (A) Noise-free image. (B) Noise corrupted image. (C) Restoration result of SM filter. (D) Restoration result of DWM filter. (E) Restoration result of MDWM filter. (F) Restoration result of MDW filter. (G) Restoration result of TVW filter. (H) Restoration result of proposed filter.

**Fig 9:**
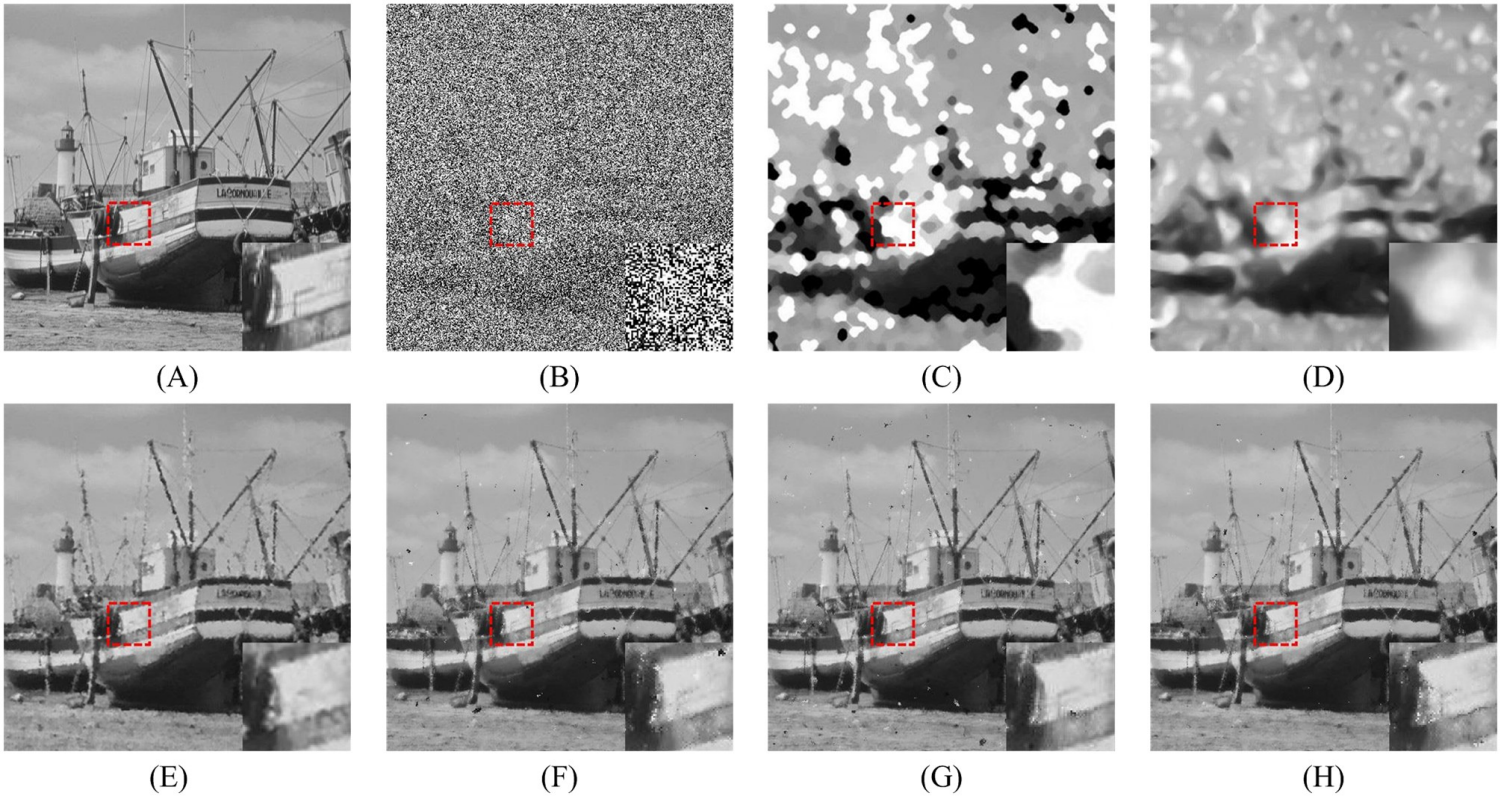
Restoration results of various filters for the Boat image with 80% noise density. (A) Noise-free image. (B) Noise corrupted image. (C) Restoration result of SM filter. (D) Restoration result of DWM filter. (E) Restoration result of MDWM filter. (F) Restoration result of MDW filter. (G) Restoration result of TVW filter. (H) Restoration result of proposed filter.

**Fig 10:**
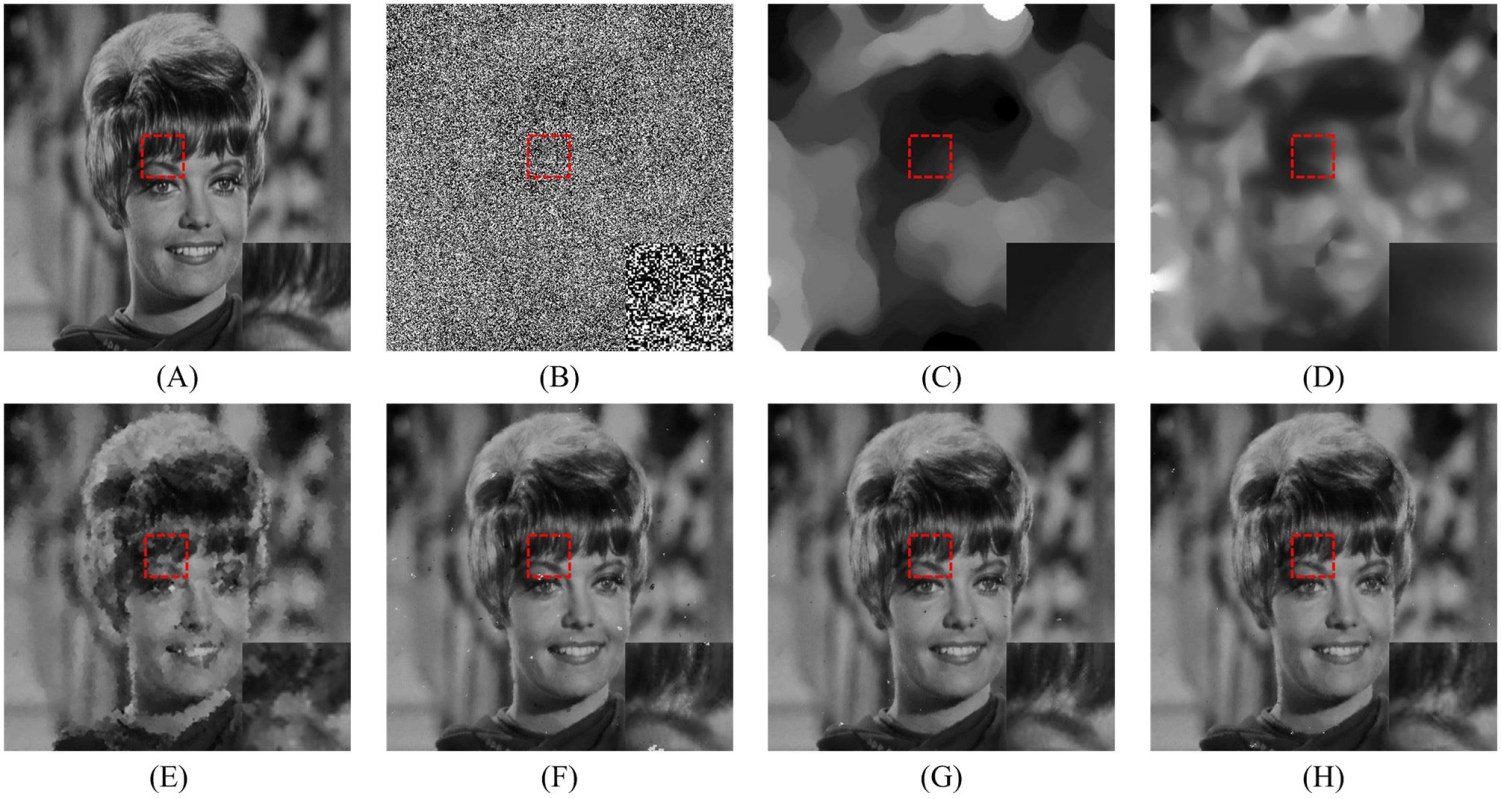
Restoration results of various filters for the Zelda image with 80% noise density. (A) Noise-free image. (B) Noise corrupted image. (C) Restoration result of SM filter. (D) Restoration result of DWM filter. (E) Restoration result of MDWM filter. (F) Restoration result of MDW filter. (G) Restoration result of TVW filter. (H) Restoration result of proposed filter.

Figs 8, 9 and 10 show the restoration results of various filters for the test images which are corrupted by salt-and-pepper noise with 80% noise density. It can be found that the SM and DWM filters fail to restore the heavy noise corrupted image. The restored images of MDWM filter suffer from a great quantity of blurred effects for edges and details. Although the DWM filter can restore image edges and details from the noise corrupted image, plenty of residual noise still exists in the restored images. The TVW filter and the proposed filter can distinguish the contours of the test images, but the TVW filter still has a few white noise and black noise in the restored images. Therefore, the above restoration results clearly indicate that the proposed filter obtains the best visual effect in terms of noise suppression and detail preservation especially when the noise density is high.

**Fig 11:**
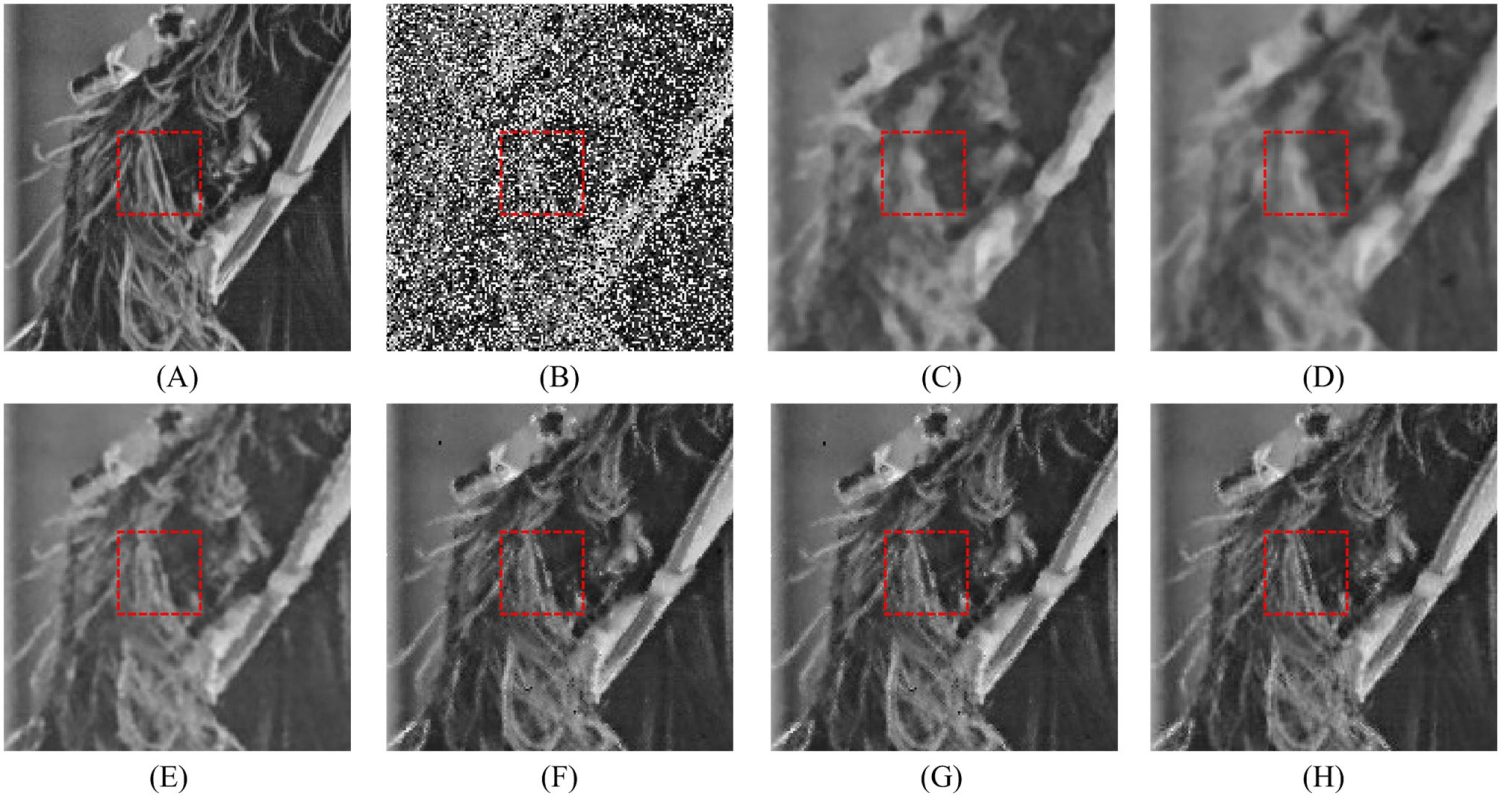
Enlarged restoration results of various filters for the details of Lena image with 50% noise density. (A) Noise-free image. (B) Noise corrupted image. (C) Restoration result of SM filter. (D) Restoration result of DWM filter. (E) Restoration result of MDWM filter. (F) Restoration result of MDW filter. (G) Restoration result of TVW filter. (H) Restoration result of proposed filter.

**Fig 12:**
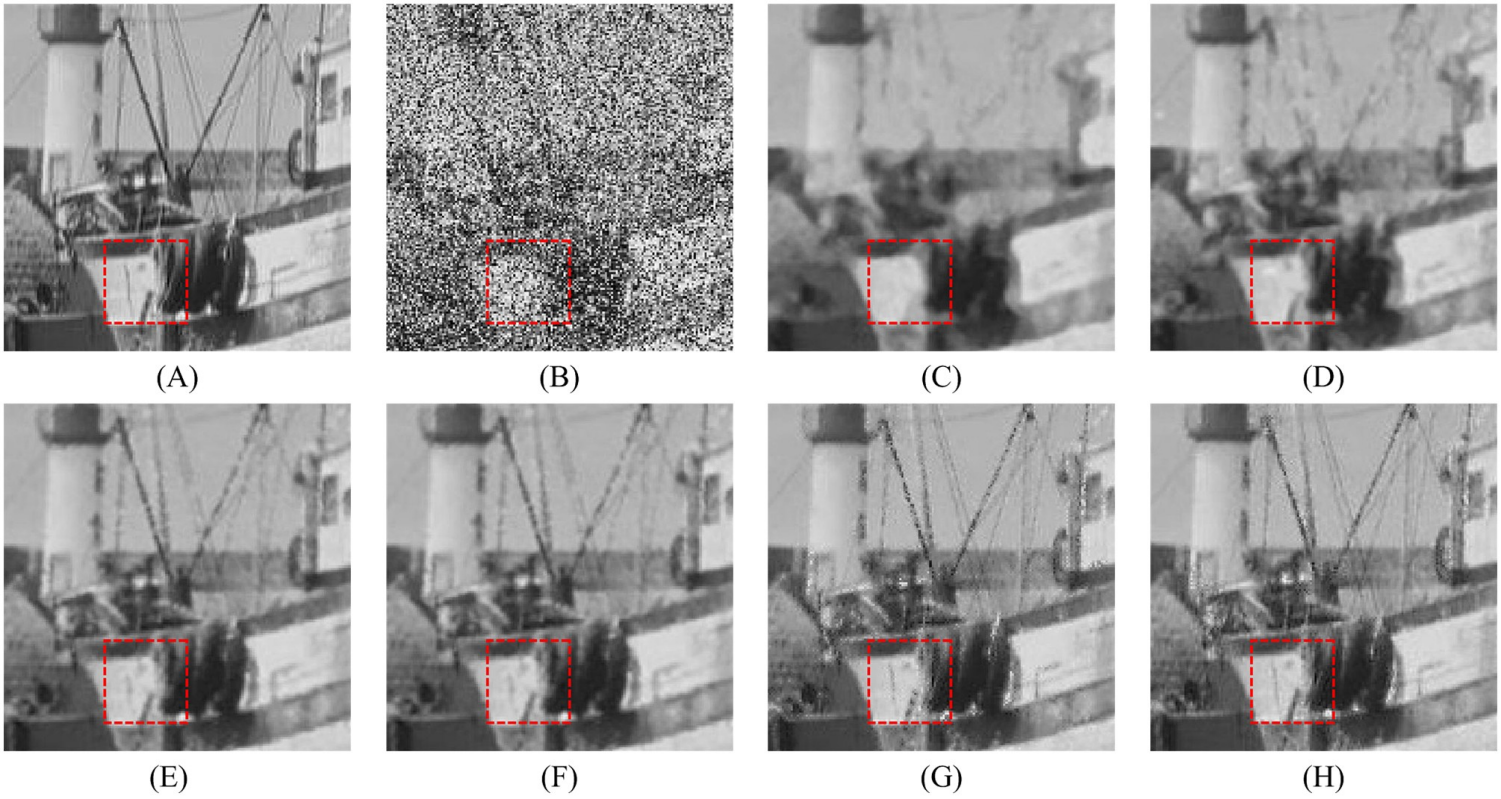
Enlarged restoration results of various filters for the details of Boat image with 50% noise density. (A) Noise-free image. (B) Noise corrupted image. (C) Restoration result of SM filter. (D) Restoration result of DWM filter. (E) Restoration result of MDWM filter. (F) Restoration result of MDW filter. (G) Restoration result of TVW filter. (H) Restoration result of proposed filter.

**Fig 13:**
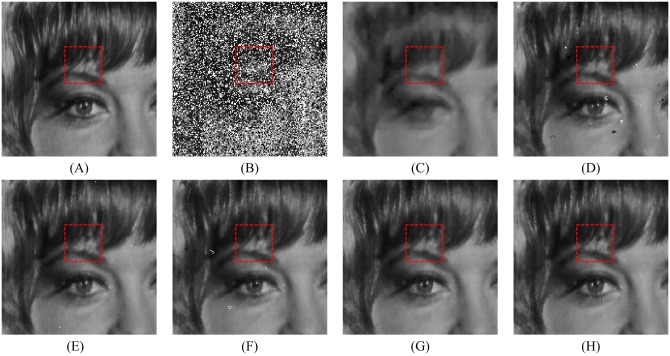
Enlarged restoration results of various filters for the details of Zelda image with 50% noise density. (A) Noise-free image. (B) Noise corrupted image. (C) Restoration result of SM filter. (D) Restoration result of DWM filter. (E) Restoration result of MDWM filter. (F) Restoration result of MDW filter. (G) Restoration result of TVW filter. (H) Restoration result of proposed filter.

**Fig 14:**
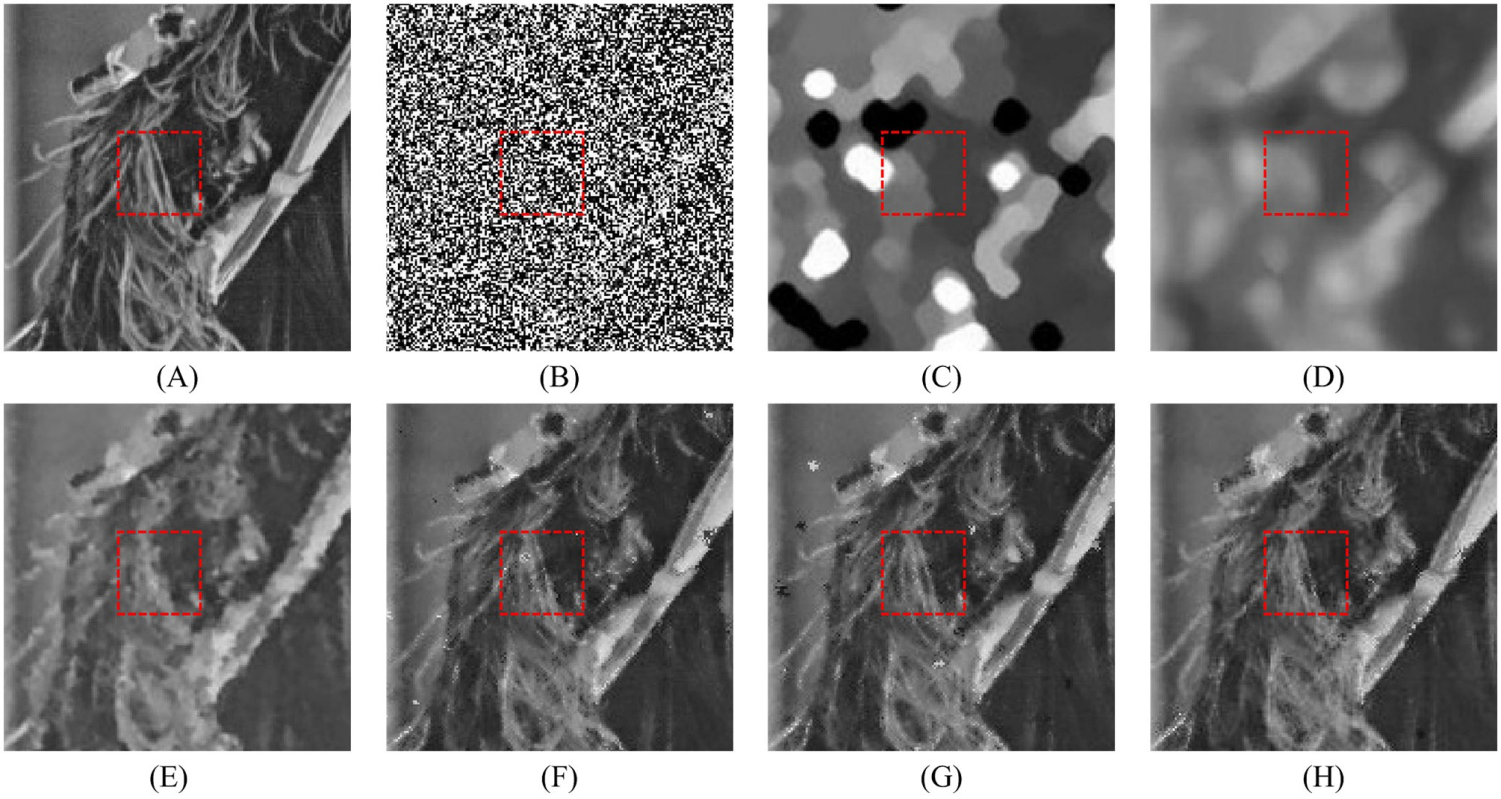
Enlarged restoration results of various filters for the details of Lena image with 80% noise density. (A) Noise-free image. (B) Noise corrupted image. (C) Restoration result of SM filter. (D) Restoration result of DWM filter. (E) Restoration result of MDWM filter. (F) Restoration result of MDW filter. (G) Restoration result of TVW filter. (H) Restoration result of proposed filter.

**Fig 15:**
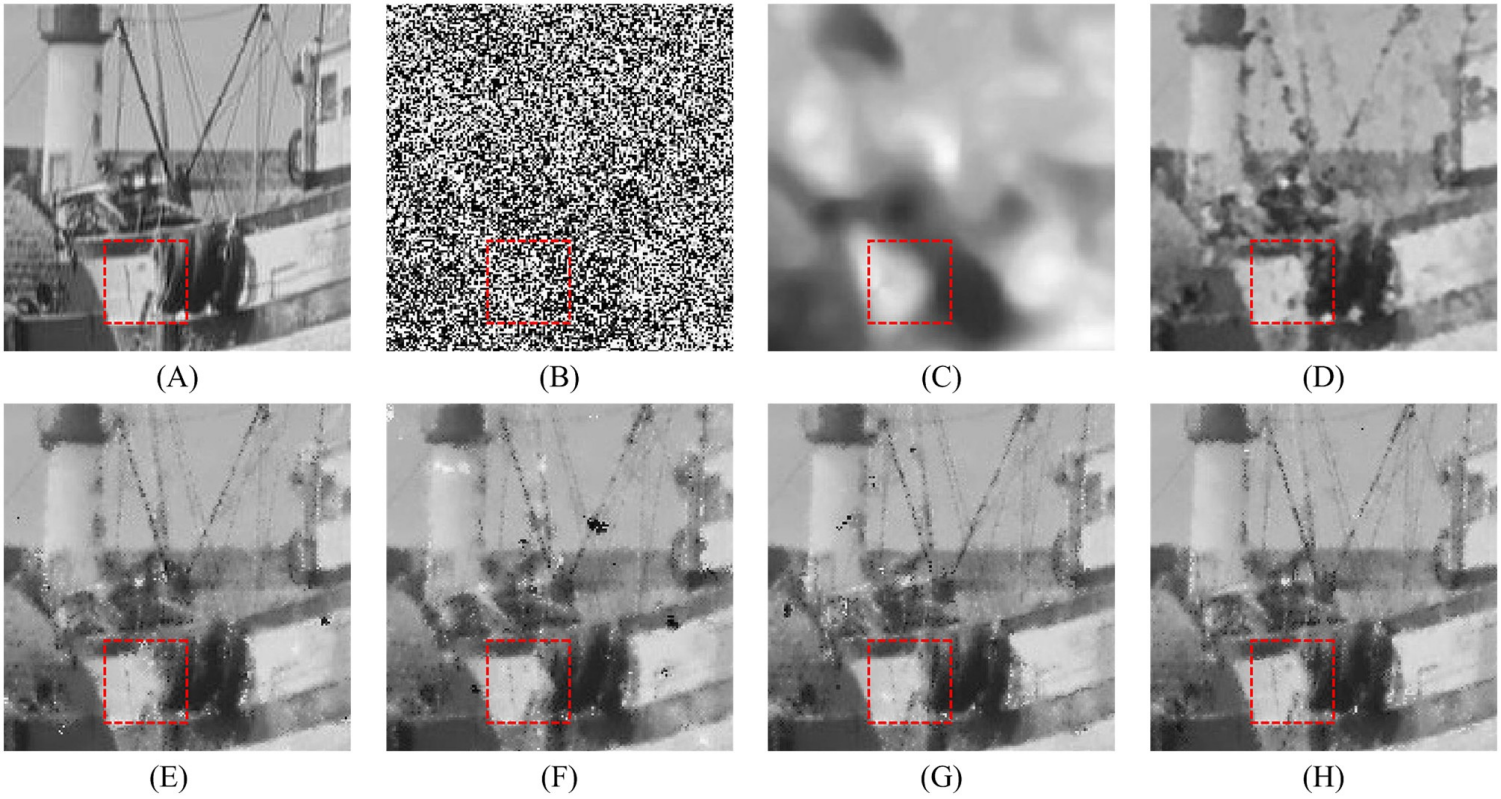
Enlarged restoration results of various filters for the details of Boat image with 80% noise density. (A) Noise-free image. (B) Noise corrupted image. (C) Restoration result of SM filter. (D) Restoration result of DWM filter. (E) Restoration result of MDWM filter. (F) Restoration result of MDW filter. (G) Restoration result of TVW filter. (H) Restoration result of proposed filter.

**Fig 16:**
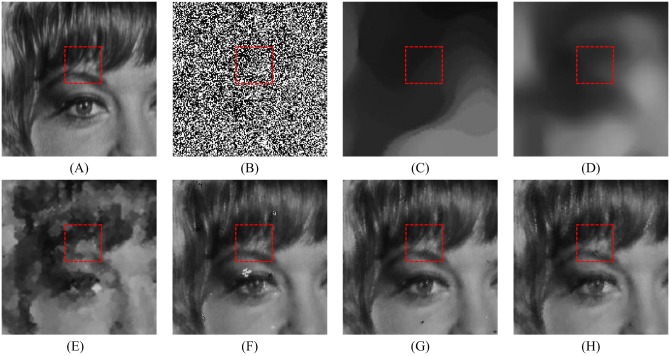
Enlarged restoration results of various filters for the details of Zelda image with 80% noise density. (A) Noise-free image. (B) Noise corrupted image. (C) Restoration result of SM filter. (D) Restoration result of DWM filter. (E) Restoration result of MDWM filter. (F) Restoration result of MDW filter. (G) Restoration result of TVW filter. (H) Restoration result of proposed filter.

The enlarged restoration results of various filters for the details of test images with 50% noise density are shown in Figs 11, 12 and 13, respectively. From Fig 11, one can observe that the details such as the tassels of Lena image, are blurred in the restored results of the SM and DWM filters. The MDWM and MDW can preserve the details, but some residual noise still exists in the restored images. The TVW filter and the proposed filter can preserve the details better than the MDWM and MDW filters. From Figs 12 and 13, the similar conclusions as Fig 11 can be obtained. The enlarged restoration results of various filters for the details of test images with 80% noise density are shown in Figs 14, 15 and 16, respectively. From Fig 14, it can be seen that the details are seriously blurred in the restored results of the SM and DWM filters. Although the details in the restored images of MDWM are blurred, the contours still can be judged from the restored images. The MDW and TVW filters can preserve the details, but some residual noise still exists in the restored images. The proposed filter can preserve the details better than the other filters. The similar conclusions as Fig 14 can be obtained from Figs 15 and 16. All of these results indicate that the proposed filter can obtains the best visual effect in terms of noise suppression and detail preservation in the cases of various noise densities.

In order to further demonstrate the effectiveness of the proposed filter, the proposed filter is compared with some state-of-the-art filters for noise removal as the adaptive weighted mean (AWM) filter [22], the adaptive decision based kriging interpolation (ADKI) filter [23], the adaptive iterative fuzzy (AIF) filter [29] and the adaptive Type-2 fuzzy (ATF) filter [31]. Table 5 presents the performance comparisons for the different filters in terms of PSNR for Lena image. It can be seen that the proposed filter can obtain the higher value of PSNR than AWM filter in the case of different noise density. Compared with the AIF and ATF filters, the proposed filter can achieve the higher value of PSNR in the case of heavy noise corruption (noise density higher than 80%). Although the ADKI filter can obtain the higher value of PSNR than the proposed filter in the case of different noise density, the operating conditions of the ADKI filter are stricter than the proposed filter. Hence, Table 5 shows that the proposed filter can perform better than some state-of-the-art filters.

**Table 5:** Quantitative comparisons of restored results in PSNR value for Lena image.

Noise density(%)	Denoising algorithms				
	AWM	AIF	ATF	ADKI	Proposed
20	36.30	39.92	40.75	41.25	39.87
50	32.62	34.10	34.88	35.85	34.02
80	27.67	28.84	28.92	30.12	29.45

Table 6 presents the performance comparisons of the proposed filter in error rate for the test images. It can be seen that the error rate of the proposed filter increases with the noise density. However, in the case of low noise corruption (noise density less than 30%), the error rate of the proposed filter does not exceed 30. And in the case of medium noise corruption, such as 40%-70% noise density, the error rate of the proposed filter is less than 65. As to the cases of heavy noise corruptions (noise density higher than 80%), the error rate of the proposed filter is less than 85. Therefore, Table 6 indicates that the proposed filter is able to effectively and accurately detect the salt-and-pepper noise.

**Table 6:** Performance comparisons of the proposed filter in error rate for the test images.

Image	Noise density(%)								
	10	20	30	40	50	60	70	80	90
Lena	8.45	17.06	25.62	34.31	42.98	51.95	60.63	70.49	80.82
Boat	9.06	17.72	26.31	34.98	43.56	52.63	61.25	71.14	81.45
Zelda	7.63	16.32	25.03	33.75	42.30	51.28	60.02	69.87	80.16

## 4 Conclusion

A new two-stage filter for removing salt-and-pepper noise using noise detector based on characteristic difference parameter and adaptive directional mean filter is proposed in this paper. There are three main contributions of the proposed filter. The first one is that the proposed filter introduces the noise detector based on the characteristic difference parameter and gray level extreme to effectively and accurately identify the noise corrupted pixels. The second one is that the proposed filter introduces a restoration scheme to secondly restore the noise corrupted pixels after primary filtering by two different restoration skills. The third one is that a new adaptive directional mean filter is developed in the restoration scheme which can effectively remove noise in the case of various noise densities meanwhile preserve some important edges and details especially at the high noise density. Experimental results on a series of images with varying noise densities show that the proposed method can perform better than many other existing main filters in terms of image denoising and detail preservation.
